# Molecular imaging of Toll-like receptor 4 detects ischemia-reperfusion injury during intussusception

**DOI:** 10.18632/oncotarget.23609

**Published:** 2017-12-22

**Authors:** Zhang-Chun Hu, Ya-Lan Tan, Shun-Gen Huang, Peng Pan, Xiao-Bo Liu, Jian Wang, Wan-Liang Guo

**Affiliations:** ^1^ Radiology Department, Children's Hospital of Soochow University, Suzhou, 215003, China; ^2^ General Surgery Department, Children's Hospital of Soochow University, Suzhou, 215003, China

**Keywords:** intussusception, ischemia-reperfusion, TLR4, in vivo imaging, PbS quantum dots

## Abstract

We investigated the expression of Toll-like receptor 4 (TLR4) in the acute phase of intestinal I/R injury during intussusception and evaluated whether anti-TLR4 antibody-conjugated lead sulfide quantum dots (TLR4-PbS QDs) could be used to detect and monitor the injury. We first established a mouse model of I/R injury during intussusception. TLR-PbS QDs were then intravenously administered to intestinal I/R injured mice and visualized using whole-body fluorescence imaging in the second near-infrared window (NIR-II). Immunohistochemical analysis of intestinal tissue from the mice revealed that TLR4 expression was higher in the I/R injury group than the control and TAK-242 groups (5.189 ± 2.482, 1.186 ± 1.171, and 2.400 ± 0.857, respectively, *P* < 0.05). NIR-II fluorescence intensity was also higher in the I/R injury group than in the control and TAK-242 groups (86.415 ± 10.955, 38.975 ± 8.619, and 71.977 ± 3.838, respectively; *P* < 0.05). Thus, anti-TLR4-PbS QDs bound to TLR4 on the cell membranes of intestinal epithelial cells with high specificity *in vitro* and *in vivo*. These results indicate that TLR4 promotes intestinal I/R injury during intussusception and that the injury can be noninvasively imaged using TLR4-PbS QDs.

## INTRODUCTION

Intussusception is a condition in which part of the intestine slides into an adjacent section [1]. It is one of the most common causes of bowel obstruction in children. Intestinal ischemia-reperfusion (I/R) injury can occur during intussusception. Early-stage I/R injury can result in mucosal erosion and hemorrhagic ulceration. These complications can frequently be reversed by treatment with vasodilators [2].

Inflammatory cytokines promote I/R injury during intussusception. Toll-like receptor 4 (TLR4) is a member of the Toll-like receptor (TLR) family of cell surface receptors. It functions in pathogen recognition and activation of the inflammatory response. Binding of ligands to TLR4 induces activation of the p38 mitogen-activated protein kinase (MAPK) pathway, which regulates the inflammatory immune response, cell survival, and cell death [3]. Several studies have demonstrated that TLR4 is involved in lung and intestinal I/R injury, but the role of TLR4 in has not been elucidated [4–6].

Fluorescence imaging in the second near-infrared window (NIR-II; 1,000–1,700 nm) provides enhanced soft-tissue contrast as well as better anatomical resolution and improved depth of penetration than conventional fluorescence imaging [7–10]. Several studies have demonstrated the feasibility of *in vivo* imaging using fluorescent or radiolabeled antibodies, receptors, and peptides [11–15]. For example, *in vivo* imaging using contrast-enhanced ultrasound and targeted microbubbles was used to evaluate therapeutic response in inflammatory bowel disease. Additionally, fluorescent antibodies against tumor necrosis factor were evaluated as molecular imaging agents in Crohn's disease [16, 17].

In this study, we investigated the expression of TLR4 in the acute phase of intestinal I/R injury during intussusception. Additionally, we evaluated whether anti-TLR4 antibody-conjugated lead sulfide quantum dots (TLR4-PbS QDs) could be used to detect and monitor I/R injury during intussusception.

## RESULTS

### Ultraviolet-visible near-infrared absorption and photoluminescence spectra of TLR4 antibody-conjugated QDs

**Figure 1:**
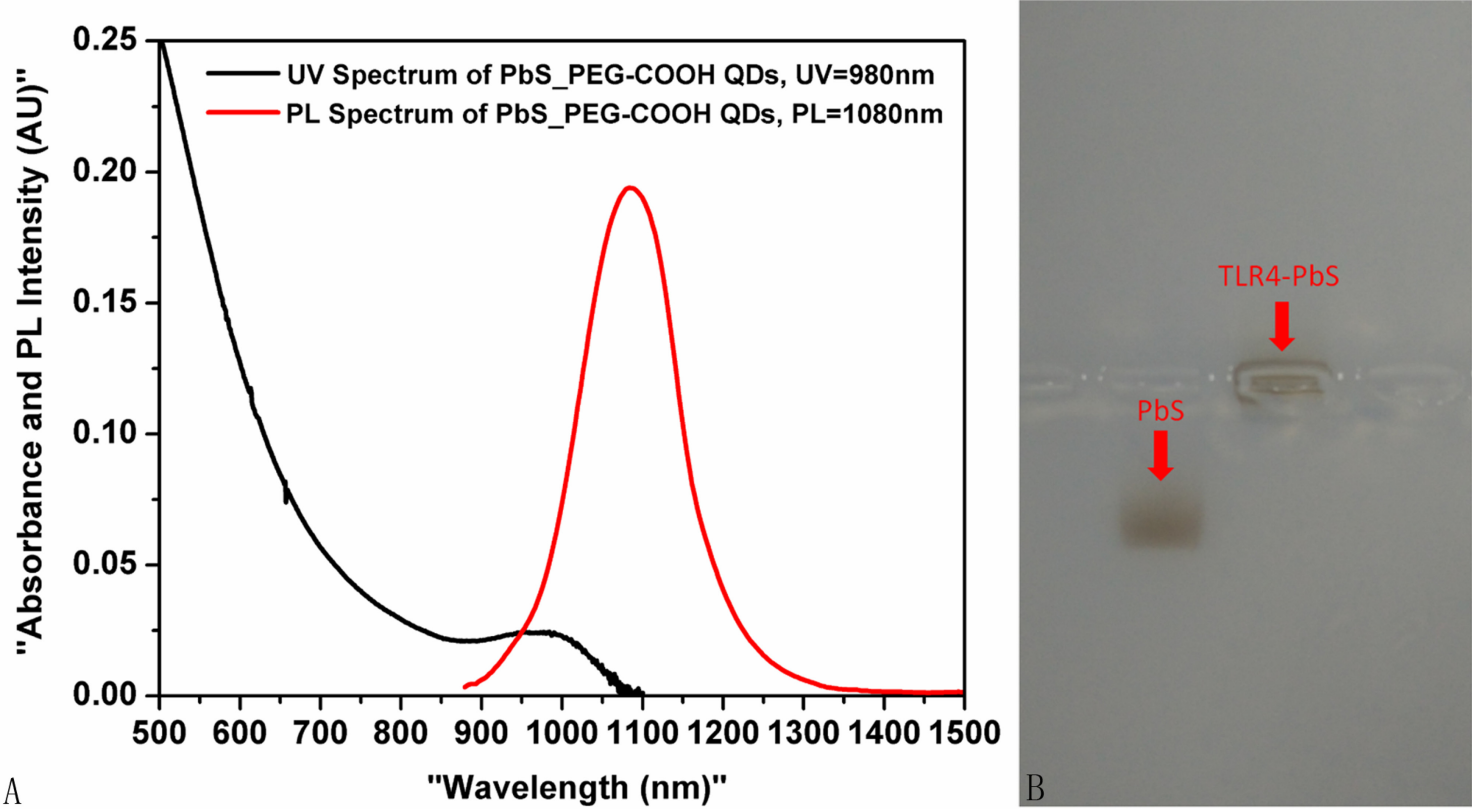
(**A**) UV-Vis-NIR absorption and photoluminescence spectra of the TLR4-PbS QDs. (**B**) Characterization of the TLR4-PbS QDs using agarose gel electrophoresis.

Ultraviolet-visible near-infrared (UV-Vis-NIR) absorption and photoluminescence spectra of the TLR4 antibody-labeled QDs revealed a peak at 1,080 nm. The first absorption peak appeared at 980 nm, with a full width at half maximum (FWHM) of 137 nm (Figure 1A).

### Characterization of TLR4-PbS QDs by agarose gel electrophoresis

Agarose gel electrophoresis indicated that the TLR4-PbS QDs were larger and migrated more slowly than the unconjugated PbS QDs (Figure 1B).

### Development of a mouse model of intestinal I/R injury during intussusception

We established a mouse model of I/R injury during intussusception (*n* = 12). Soft tissue masses were visible in the intestines of all 12 mice consistent with bowel obstructions. Small obstructions, and areas of vascular compromise and venous congestion, were observed within 60 min of the onset of intussusception. We then reduced the intussusception and allowed reperfusion to occur for 90 min. Areas of vascular compromise and small bowel obstruction worsened following reperfusion. Histological analysis demonstrated an increase in inflammation and the number of necrotic areas in the I/R injury compared to control group.

### *In vivo* NIR-II imaging

**Figure 2:**
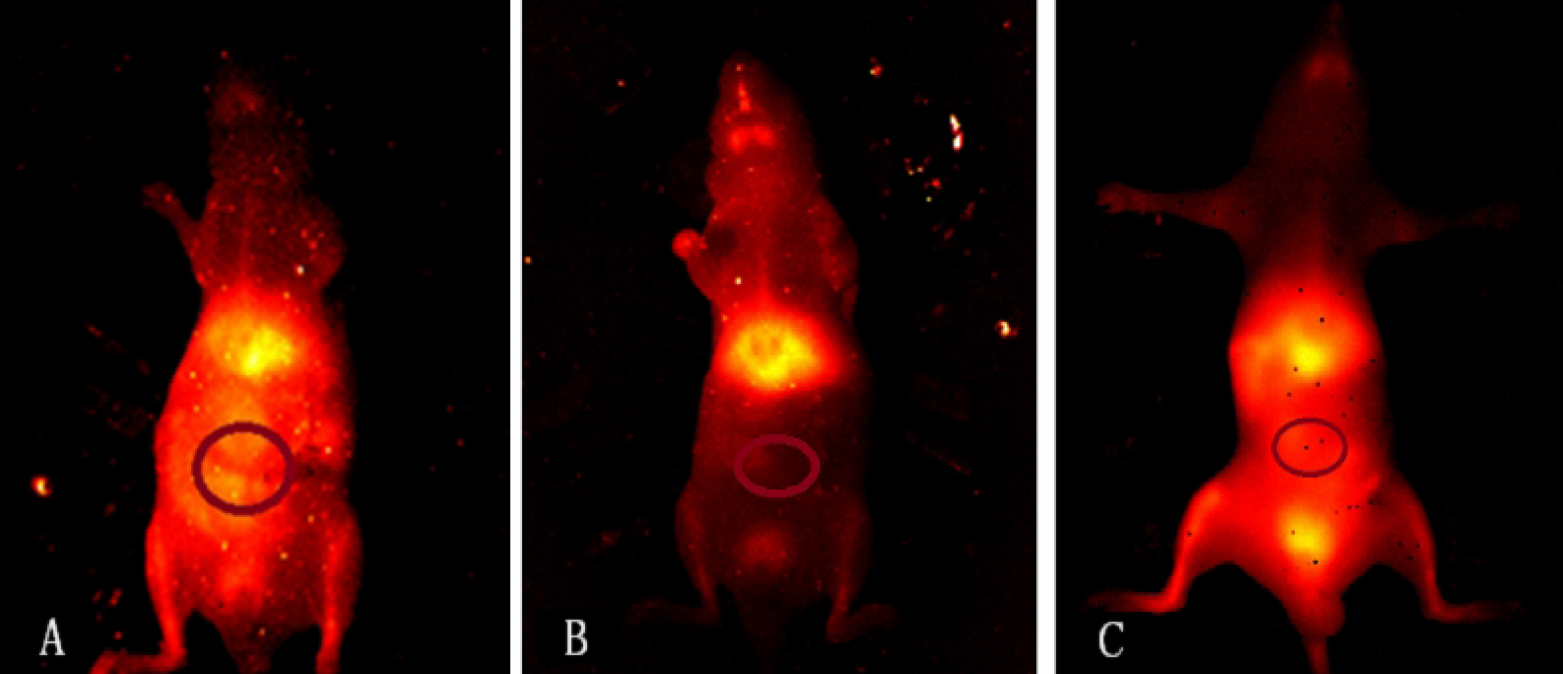
NIR-II fluorescence images of living mice 30 min after administration of an 0.15 mL bolus of the TLR4-PbS QDs in the intestinal I/R injury (**A**), control (**B**), and TAK-242 (**C**) groups.

**Figure 3:**
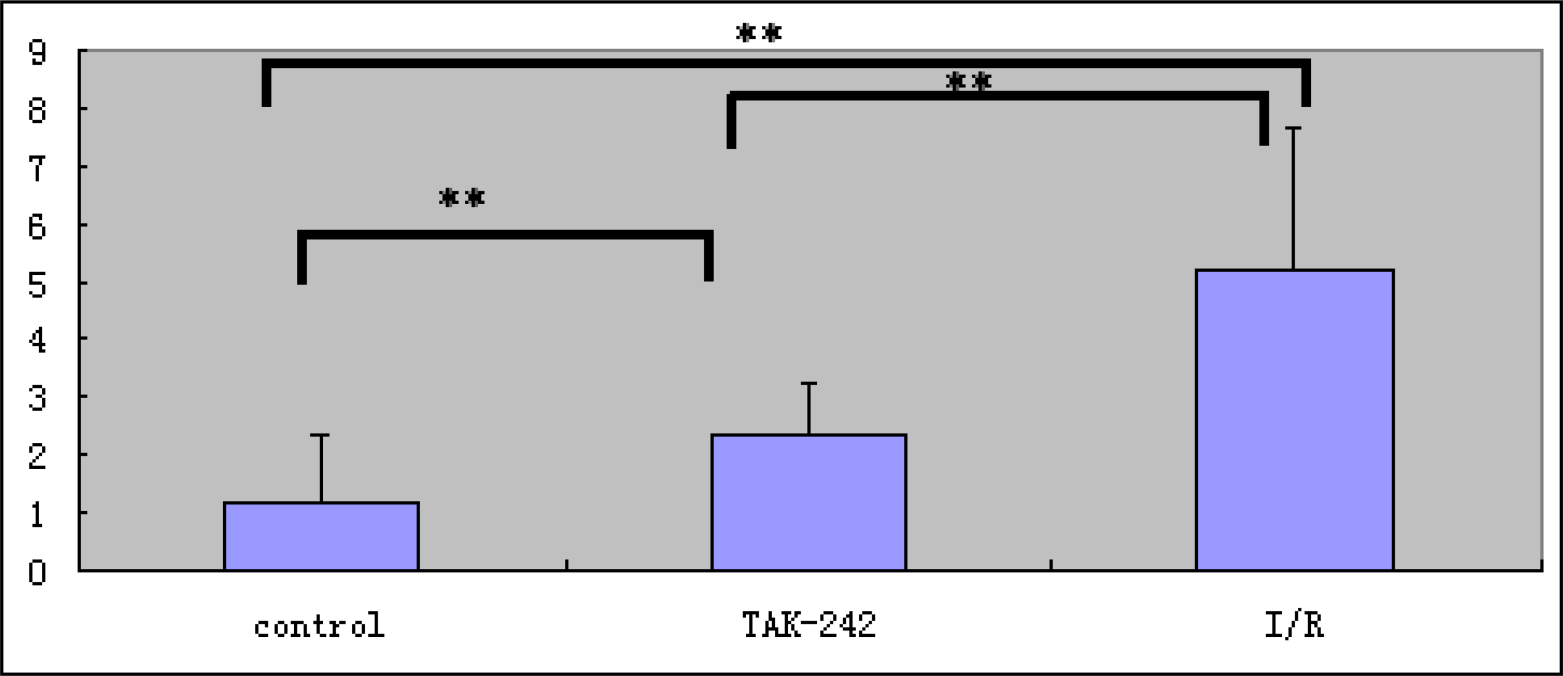
Analysis of the short-term retention of TLR4-PbS QDs in areas of intestinal damage in the I/R injury, control, and TAK-242 groups (^**^ = *P* < 0.05)

Bright fluorescence was observed in the reticuloendothelial system including the liver and spleen. Bright fluorescence was also observed in the intestine in the intussusception I/R injury group (Figure 2). Higher fluorescence was observed in the TLR4-PbS QD compared to the control and TAK-242 groups (86.415 + 10.955, 38.975 ± 8.619, and 71.977 + 3.838, respectively) (*P* < 0.05, Figure 3). Increased fluorescence was observed in regions of I/R injury 15 min after injection of the TLR4-PbS QDs. The signal reached a maximum peak after 30 min, and then decreased after 60 min (Supplementary Figures 1 and 2).

### *Ex vivo* imaging of TLR4 expression

**Figure 4:**
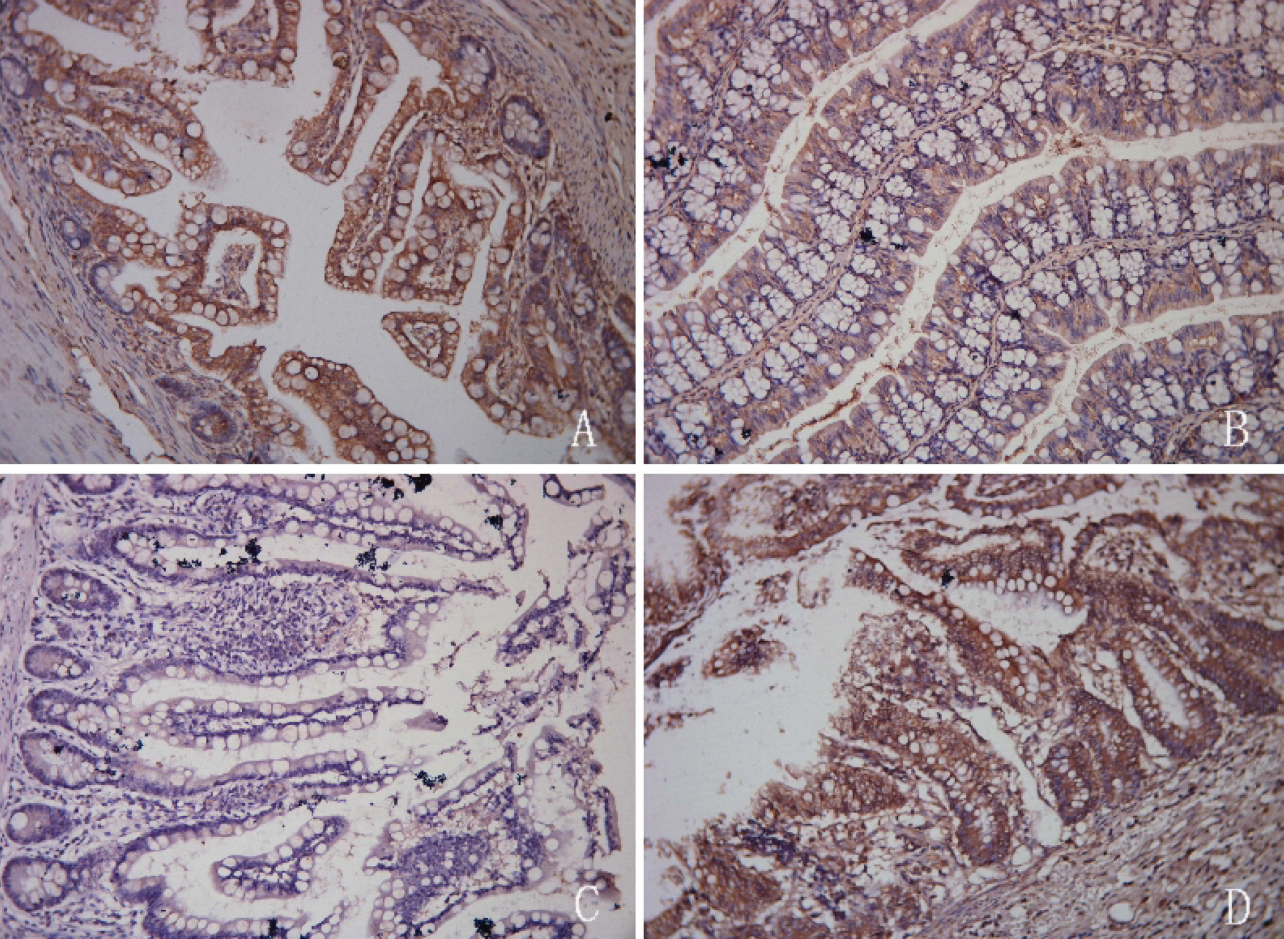
Immunohistochemical analysis showing higher TLR4 expression in the intestinal I/R injury (**A**) compared to control (**B**), negative control (**C**), and TAK-242 (**D**) groups.

**Figure 5:**
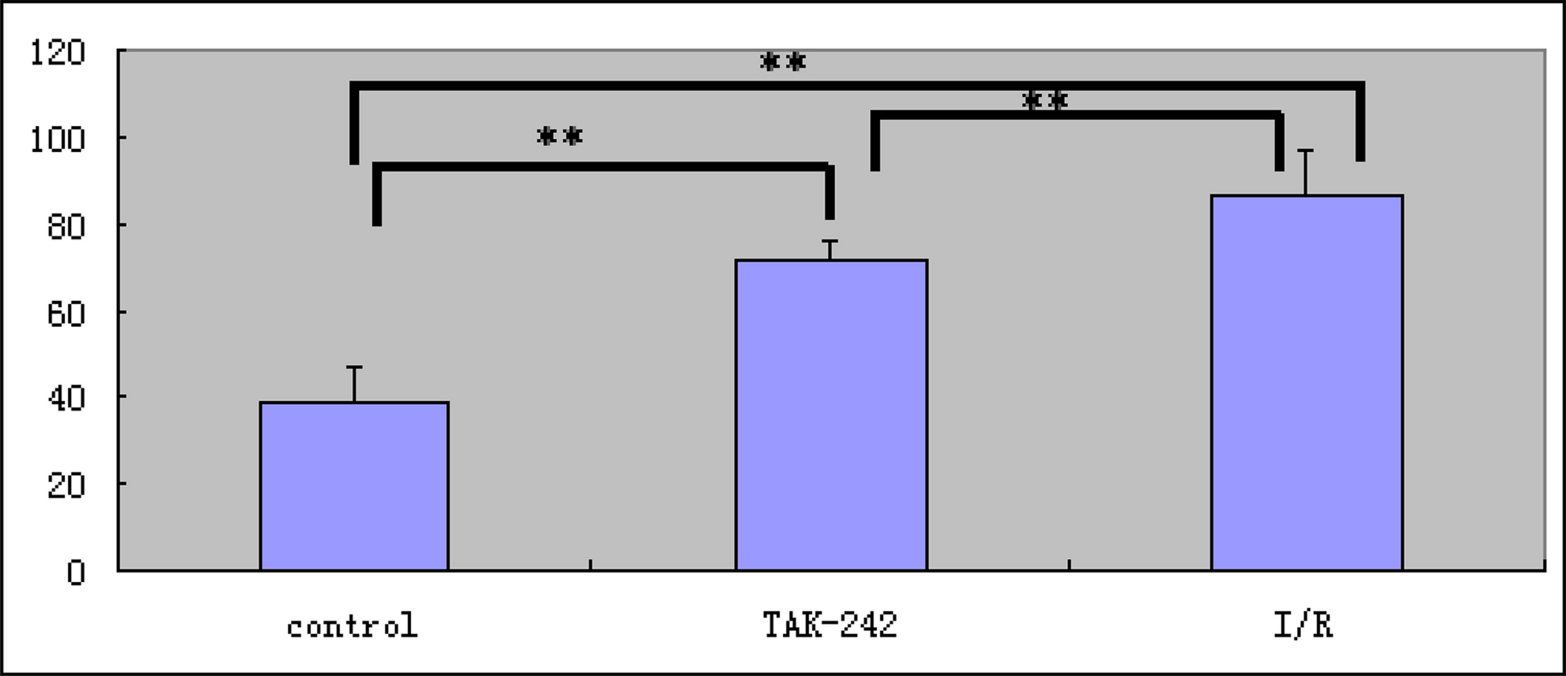
Immunohistochemical analysis of TLR 4 expression (^**^ = *P* < 0.05)

**Figure 6:**
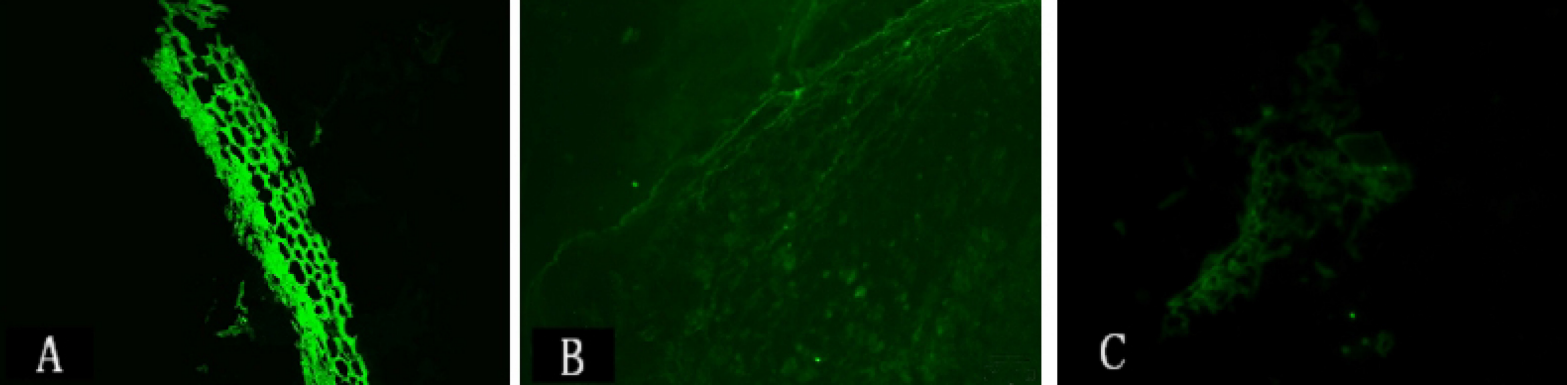
*Ex vivo* NIR-II immunofluorescence imaging showing higher TLR4 expression in intestinal tissue samples from the intestinal I/R injury (**A**) compared to control (**B**) and TAK-242 (**C**) groups (magnification: ×400).

**Figure 7:**
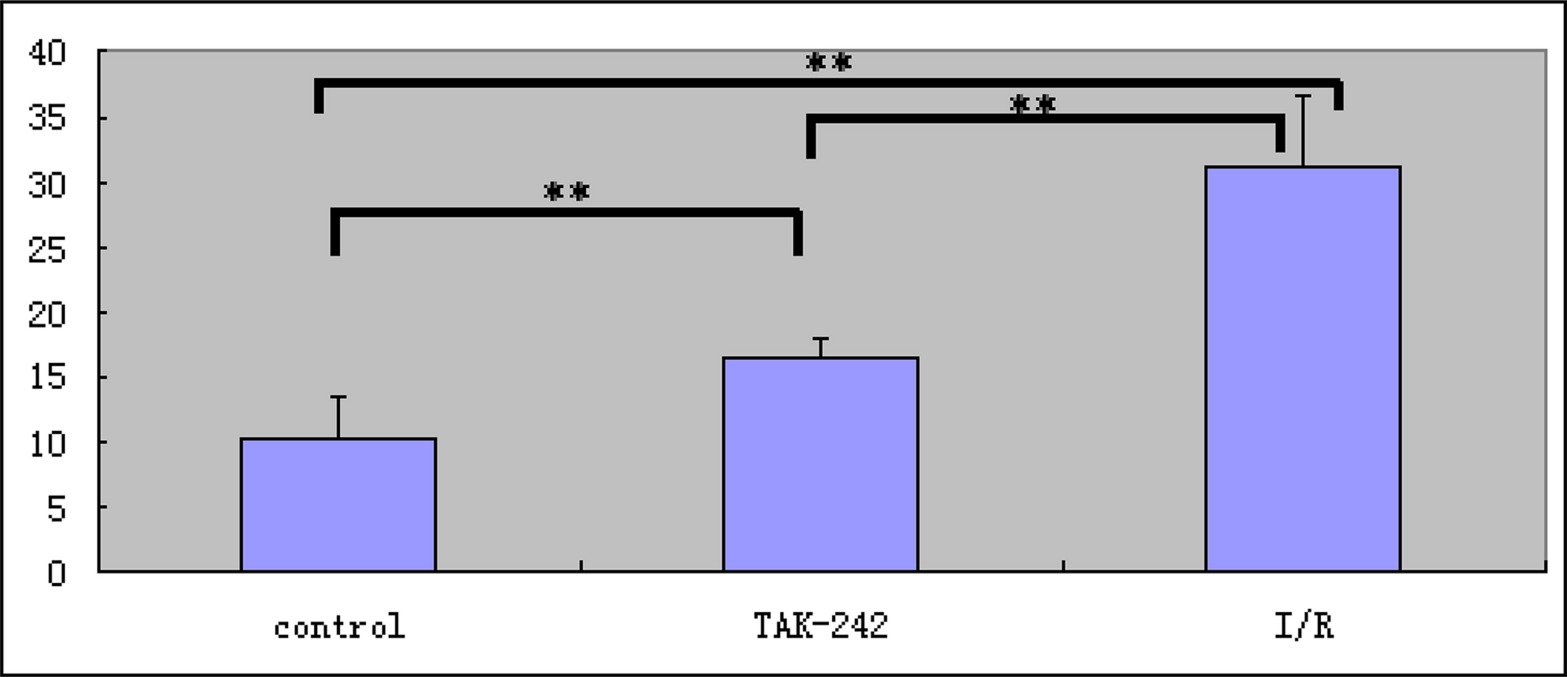
Analysis of TLR4 expression in intestinal tissue using *ex vivo* NIR-II immunofluorescence imaging (^**^ = *P* < 0.05)

**Figure 8:**
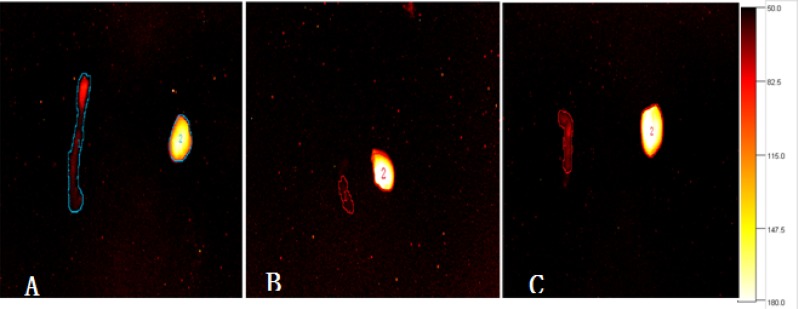
*Ex vivo* NIR-II fluorescence images of liver and intestinal tissue from mice 30 min after administration of the TLR4-PbS QDs Higher TLR4 expression was observed in the intestinal I/R injury (**A**) compared to control (**B**) and TAK-242 (**C**) groups.

Immunohistochemical analysis indicated that intestinal epithelial cells had a characteristic columnar appearance. TLR4 was predominantly expressed on the cell membranes (Figure 4). The MQSs for TLR4 expression in intestinal epithelial cells were 5.189 ± 2.483, 1.186 ± 1.171, and 2.400 ± 0.857 in tissue samples from the I/R injury, control, and TAK-242 groups, respectively (*P* < 0.05, Figure 5). Immunofluorescence analysis demonstrated that TLR4 expression was higher in the I/R injury compared to control and TAK-242 groups (31.127 ± 5.482, 10.215 ± 3.255, and 16.500 ± 1.332, respectively, *P* = 0.0005, Figures 6 and 7). *Ex vivo* NIR-II imaging also demonstrated increased fluorescence in tissue samples from the I/R injury compared to control and TAK-242 groups (Figure 8). Immunohistochemical analysis indicated that p38 MAPK expression was higher in the I/R injury compared to control and TAK-242 groups (Supplementary Figure 3). The MQSs for p38 MAPK in intestinal epithelial cells were 8.712 ± 0.920, 2.109 ± 0.109, and 5.447 ± 0.526 in tissue samples from the I/R injury, control, and TAK-242 groups, respectively (*P* < 0.0001, Supplementary Figure 4).

### *In vitro* model of intestinal I/R injury

**Figure 9:**
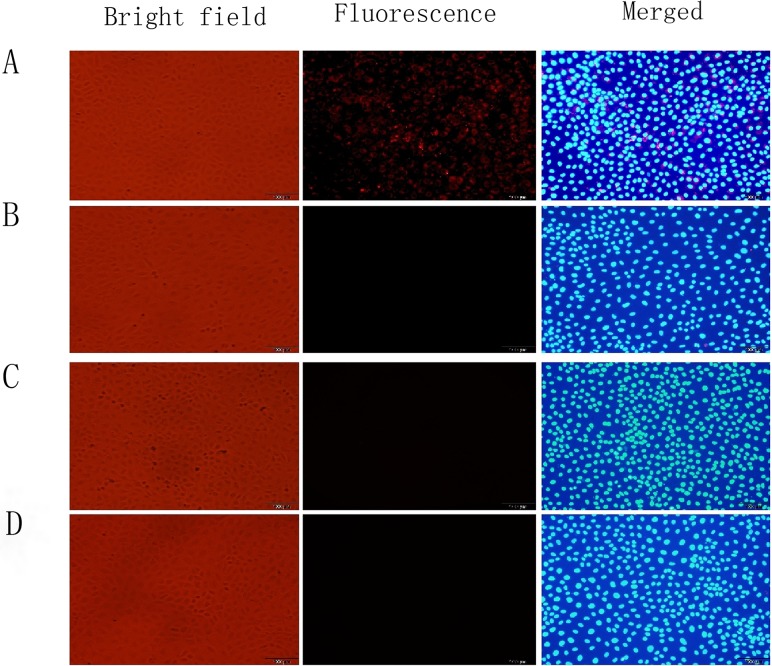
Immunofluorescence analysis of TLR4-CdSe QD binding activity in an *in vitro* model of intestinal I/R injury Positive TLR4 expression on the cell membranes of intestinal epithelial cells in the I/R injury model (**A**). No staining of TLR4 was observed in the QD (**B**), control (**C**), and TLR4-treated (**D**) groups.

We evaluated the specificity of TLR4-CdSe QDs for TLR4 in an *in vitro* cell culture model of intestinal I/R injury. We observed a stronger fluorescence signal in the TLR4-CdSe QD compared to control (untreated) and CdSe QD groups. We then pretreated the cells with TLR4 for 30 min and evaluated the fluorescence in the I/R injury compared to control group (Figure 9). These results indicated that the TLR-CdSe QDs bound TLR4 with high specificity.

## DISCUSSION

We investigated TLR4 expression in intestinal I/R injury during intussusception. Our data indicated TLR4 expression was higher in areas of intestinal I/R injury than in the normal intestinal epithelium. Using NIR-II fluorescence imaging, we determined that TLR4-PbS QDs bound TLR4 on the surface of intestinal epithelial cells with high specificity both *in vitro* and *in vivo*. The TLR4-PbS QDs localized to areas of intestinal damage in a mouse model of I/R injury during intussusception and in human tissue from pediatric intussusception patients. Thus, TLR4-PbS QDs could be used to detect early-stage I/R injury during intussusception. Collectively, our data indicate that TLR4 may have an important role in activating the inflammatory response during intussusception.

The restoration of blood supply to ischemic tissues can result in activation of an acute inflammatory response leading to additional tissue damage. Previous studies have demonstrated that TLR4 can activate the MAPK and NF-kB pathways, which are critical regulators of the inflammatory response, cell survival, and apoptosis [3]. We previously observed high p38 MAPK expression in response to I/R injury [18]. Additionally, Ben et al. demonstrated that TLR4 plays an important role in acute lung injury following intestinal ischemia [6].

Intestinal I/R injury during intussusception can result in necrosis of the bowel, lung injury, and multiple organ dysfunction syndrome [19]. Currently, the diagnosis of intussusception is based on a combination of serum biochemical markers, ultrasound imaging, computed tomography, magnetic resonance imaging, positron emission tomography (PET), or invasive endoscopic techniques [20]. The early detection of intestinal I/R injury using non-invasive molecular imaging may improve prognosis and treatment efficacy [15].

Antibodies conjugated to fluorescent probes such as QDs have been developed as non-invasive molecular imaging tools [15, 21]. Li et al. reported that fluorescent imaging of NIR-II probes is advantageous because it can achieve high contrast and facilitate deep-tissue imaging [20, 22]. Thackeray et al. developed a gallium-68 pentixafor to image the chemokine receptor CXCR4 in the myocardium following myocardial infarction using PET [23].

We investigated whether TLR4-PbS QDs could be used to detect and monitor intestinal I/R during intussusception. The TLR4-PbS QDs localized to areas of tissue damage in a mouse model of I/R injury during intussusception. The TLR4 QDs bound to TLR4 with high specificity both *in vitro* and *in vivo*. Our findings are consistent with those of two previous studies [15, 24]. Li et al. demonstrated that Ag2S QDs allowed *in vivo* monitoring of lymphatic drainage and vascular networks, and enabled targeted delivery of nanodrugs that prevent osteolysis to metastatic bone tumors. The Ag2S QDs demonstrated improve tissue penetration depth and enhanced spatiotemporal resolution [25, 26]. We have demonstrated that TLR4-PbS QDs may be useful for ultrasensitive NIR-II fluorescence imaging *in vivo* [27].

Our study had several limitations. First, we did not investigate the cytotoxicity of the TLR4-PbS QDs. Additional studies are required to investigate these effects in major organs including the liver and spleen. Second, we did not explore the pharmacokinetics including clearance time of the TLR4-PbS QDs.

In summary, we have shown that TLR4 is highly expressed on the membranes of intestinal epithelial cells in areas of I/R injury during intussusception. TLR4-PbS QDs bind to TLR4 with high specificity *in vivo*. These molecular probes may be useful for high-resolution, non-invasive imaging of early-stage I/R injury during intussusception.

## MATERIALS AND METHODS

### Spectrophotometry

Photoluminescence spectra were acquired using a fiber fluorescence spectrophotometer (Ocean Optics NIR Quest and QE6500, Ocean Optics, Largo, FL, USA) equipped with a HGIL T250 250 W double-grating monochromator. Absorption spectra were acquired on a UV-Vis spectrophotometer (Agilent 8453, Agilent, Santa Clara, CA, USA). Quantum yield (QY) was calculated using the following equation:

$${Ф}_{\text{x}}=Ф\text{st}(\text{Ix}/\text{Ist})(\eta \text{x}2/\eta \text{st}2)(\text{Ast}/\text{Ax}),$$

where Ф is the quantum yield, I is the measured integrated emission intensity, η is the refractive index of the solvent, and A is the optical density. The subscript “st” refers to the standard with known quantum yield and “x” refers to the QD sample. Fluorescence spectra were measured at excitation wavelengths below 785 nm. IR-26 dye dissolved in 1,2-dichloroethane was used as a reference.

### Conjugation of an anti-TLR4 antibody to QDs

Water-soluble, NIR-emitting PbS QDs were purchased from Mesolight Inc. (Suzhou, China). The particles were coated with polyethylene glycol (PEG) modified with carboxylic acid to ensure colloidal stability and solubility in an aqueous environment. The QDs were obtained as a 6 μM stock solution dissolved in sodium borate buffer (10 mM, pH = 7.4). We conjugated the anti-TLR4 antibody to the QDs as described previously [28]. Briefly, QDs capped with PEG-COOH were dissolved in 10 mM sodium borate reaction buffer (pH 7.4) to a final concentration of 1 μM. A total of 50 μL of the TLR4 antibody (Mouse monoclonal, Abcam, USA) was then added to the QDs while vortexing. A 10 mg/mL stock solution of 1-ethyl-3-(3-dimethylaminopropyl) carbodiimide in water was prepared and 50 μL of the solution immediately added to the reaction mixture. The reaction was incubated for 2 h at room temperature with gentle mixing. Following the incubation, the solution was centrifuged at 13,000 rpm with a Microsep^TM^ Advance Centrifugal Device (YM-100KD, Pall Corporation, Port Washington, NY, USA) for 10 min to remove free antibodies. TLR4-PbS QDs were recovered in 300 μL 1× phosphate-buffered saline containing 0.05% sodium azide and stored at 4°C until use.

### Animal experiments

The study protocol was approved by the Institutional Ethics Review Committee of the Children's Hospital of Soochow University. All experiments were performed in accordance with the institutional guidelines for the use of animals and human tissue in experiments. A total of 18 nude mice (a mixture of male and female, 4–6 weeks old, 15–20 g) were included in the study. The mice were divided into experimental (*n* = 12) and control (*n* = 6) groups. To evaluate the binding specificity of the TLR4-PbS QDs for TLR4, we further divided the mice in the experimental group into I/R injury (*n* = 6) and TAK-242 (*n* = 6) groups. TAK-242 (3 mg/kg), a specific inhibitor of TLR4, was administered intraperitoneally 60 min prior to intussusception. Intussusception was established under ether inhalation anesthesia as described previously [18]. Intussusception was observed in all mice in the I/R injury and TAK-242 groups after 60 min. Reperfusion was allowed to proceed for 90 min.

### *In vivo* and *ex vivo* NIR-II fluorescence imaging

*In vivo* whole-body and *ex vivo* fluorescence imaging of organs and tissues was performed with a customized NIR-II fluorescence imaging system (Suzhou Institute of Nano-Tech and Nano-Bionics, Chinese Academy of Sciences, China) equipped with an 808 nm diode laser (880 nm long-pass filter). The distribution of the TLR4-PbS QDs in living mice was monitored in real-time and images acquired continuously (50 mW/cm^2^ power, 100 ms exposure time). TLR4-PbS QDs were administered as a 0.10–0.15 mL bolus via the tail vein. Dynamic contrast-enhanced images were obtained 1 min, 15 min, 30 min, 45 min, 60 min, and 90 min after injection. Regions of interest within the intestine were identified using markers that were placed on the skin during the intussusception procedure. Images were analyzed using MATLAB.

### *Ex vivo* validation studies

Animals were anesthetized with 10% chloral hydrate (20 mL) and then sacrificed using the cervical dislocation method. Fluorescent imaging of the TLR4-PbS QDs was performed to identify areas of intestinal I/R injury. Tissue samples were embedded in optimal cutting temperature compound and cryosectioned into 6 mm thick sections. Sections were transferred onto Superfrost Plus slides (American MasterTech). Tissue samples were stained with hematoxylin and eosin and imaged by light microscopy (Olympus CKX31/41, Tokyo, Japan). TLR4 expression was analyzed by immunohistochemistry and immunofluorescence, while p38 MAPK expression was analyzed by immunohistochemistry. Protein expression was quantified using the IPP 6.0 image analysis software (Media Cybernetics, Rockville, MD, USA). Image analysis was performed as described previously [18]. Quick scores (QSs) were calculated using the following formula: QS = percentage of positive cells × mean intensity. Average QSs were calculated for the various fields of view in each section to obtain mean QSs (MQSs).

### *In vitro* model of I/R injury

IEC-6 non-transformed rat intestinal epithelial cells (CRL-1592; EK-Biosciences, China) were maintained in Dulbecco's Modified Eagle's Medium supplemented with 10% fetal bovine serum, 2 mM glutamine, 1% penicillin, and 1% streptomycin at 37°C in an incubator with a humidified atmosphere containing 5% CO_2_/95% air. Experiments were performed on cells between passages 17–25. An *in vitro* model of intestinal I/R injury was established by culturing cells under conditions of hypoxia followed by reoxygenation. Cells were placed in a hypoxic chamber with 1% O_2_ /5% CO_2_ /94% N_2._ Reoxygenation was then allowed to occur for the indicated time periods [29]. We evaluated the binding specificity of TLR4 antibody-conjugated cadmium selenide (CdSe) QDs (TLR4-CdSe QDs) for TLR4 by comparing the fluorescence intensity in cells treated with TLR4-CdSe QDs to those treated with CdSe QDs using confocal microscopy. We then pretreated the I/R injury cell model with TLR4 for 30 min at room temperature, and compared the fluorescence intensity of the TLR4-CdSe QDs to unconjugated CdSe QDs.

### Statistical analysis

All data were analyzed using the SAS v8.1 software (SAS Institute, Cary, NC, USA). The data are reported as the mean ± standard deviation. Differences between groups were assessed using Student-Newman-Keuls and Wilcoxon signed-rank tests. *P* values < 0.05 were considered statistically significant.

### Ethics statement

The study protocol was approved by the Institutional Ethics Review Committee of the Children's Hospital of Soochow University. All experiments were performed in accordance with the institutional guidelines regarding the care and use of animals and human tissues in experiments.

## SUPPLEMENTARY MATERIALS FIGURES



## Abbreviations

I/R: Ischemia-reperfusion
TLR: Toll-like receptor
MAPK: mitogen-activated protein kinase
PET: positron emission tomography
NIR-II: second near-infrared window
QD: Quantum dot
