# Supplementary material for: Molecular imaging of Toll-like receptor 4 detects ischemia-reperfusion injury during intussusception
Source: Oncotarget. 2017 Dec 22;9(8):7882–90. doi: 10.18632/oncotarget.23609 (PMC5814266; doi:10.18632/oncotarget.23609)
Supplement: Supplementary file 1 [file oncotarget-09-7882-s001.pdf]

Molecular imaging of Toll-like receptor 4 detects ischemia-reperfusion injury during intussusception

SUPPLEMENTARY MATERIALS

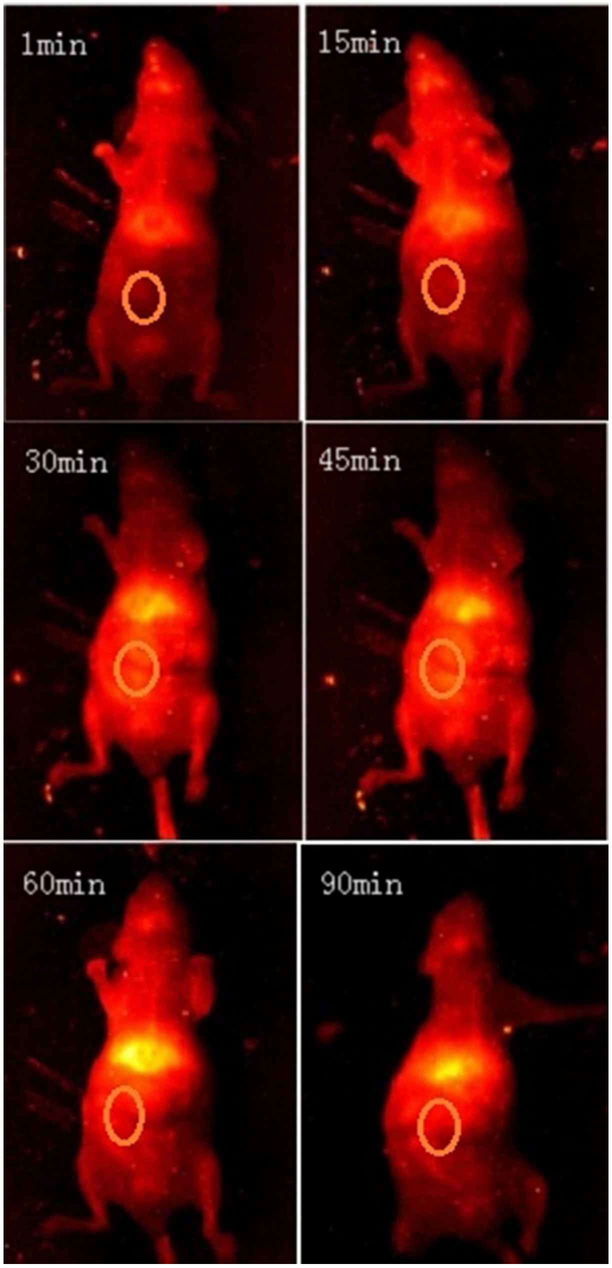

Supplementary Figure 1: NIR-II fluorescence images of living mice at the indicated time points following administration of an 0.15 mL bolus of TLR4-PbS QDs.

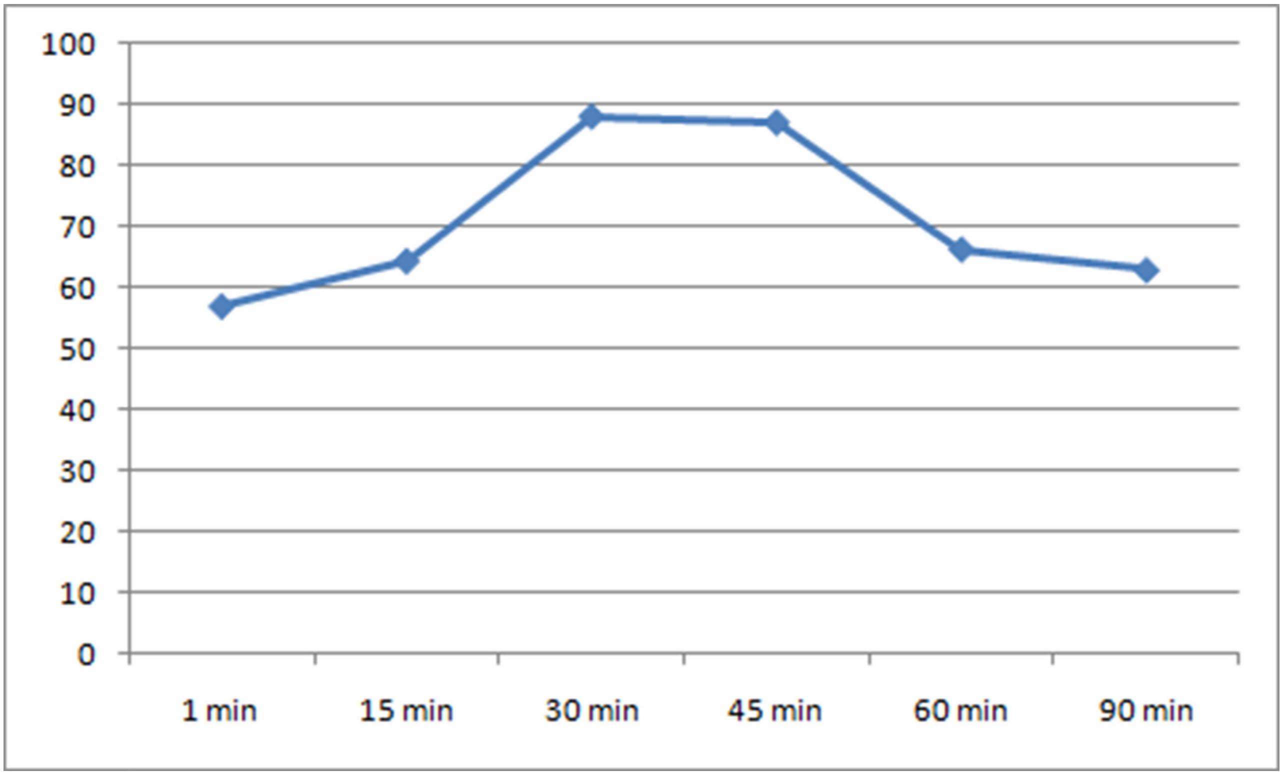

Supplementary Figure 2: Quantitative analysis of the short-term retention of the TLR4-PbS QDs in areas of intestinal damage at different time points.

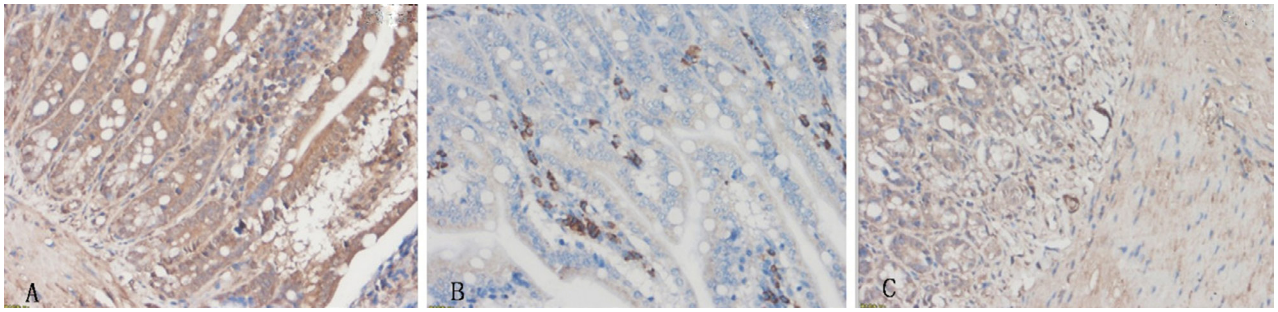

Supplementary Figure 3: Immunohistochemical analysis showing higher p38 MAPK expression in the intestinal I/R injury (**A**) compared to control (**B**), and TAK-242 (**C**) groups.

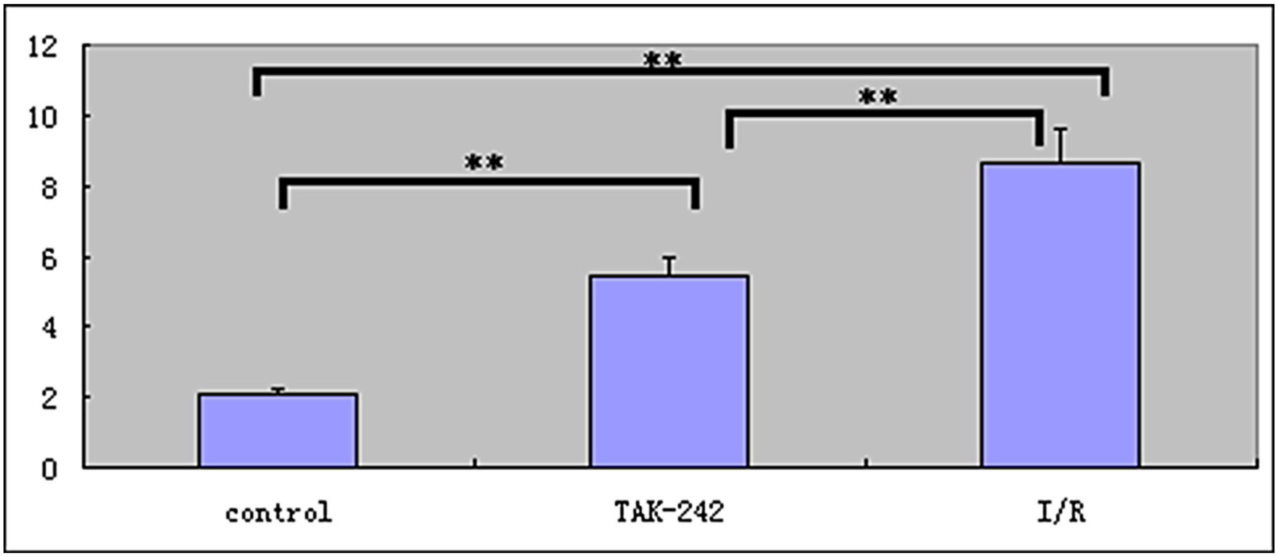

Supplementary Figure 4: Immunohistochemical analysis of p38MAPK expression (= $P < 0.05$).**
